# Gitelman syndrome with normocalciuria – a case report

**DOI:** 10.1186/s12882-022-02782-y

**Published:** 2022-05-04

**Authors:** Mariusz Flisiński, Ewa Skalska, Barbara Mączyńska, Natalia Butt-Hussaim, Agnieszka Sobczyńska-Tomaszewska, Olga Haus, Jacek Manitius

**Affiliations:** 1 Department of Nephrology, Hypertension and Internal Medicine, Collegium Medicum in Bydgoszcz, Nicolaus Copernicus University in Toruń, ul. Curie-Skłodowskiej 9, 85-094 Bydgoszcz, Poland; 2 Student’s Scientific Association of Department of Nephrology, Hypertension and Internal Medicine, Collegium Medicum in Bydgoszcz, Nicolaus Copernicus University in Toruń, ul. Curie-Skłodowskiej 9, 85-094 Bydgoszcz, Poland; 3 Medgen - MedGen Diagnostic Laboratory, MedGen Medical Center, ul. Wiktorii Wiedeńskiej 9a, 02-954 Warsaw, Poland; 4 Department of Clinical Genetics, Collegium Medicum in Bydgoszcz, Nicolaus Copernicus University in Toruń, ul. Curie-Skłodowskiej 9, 85-094 Bydgoszcz, Poland

**Keywords:** Gitelman syndrome, Tubulopathy, Urinary calcium excretion, Normocalciuria, Aldosterone, A case-report

## Abstract

**Background:**

Gitelman Syndrome (GS) is a hereditary tubulopathy associated with a biallelic inactivating mutations of the *SLC12A3* gene encoding the thiazide-sensitive sodium-chloride cotransporter (NCCT). The typical clinical manifestation is a hypokalemic metabolic alkalosis with significant hypomagnesemia, and low urinary calcium excretion. Hypocalciuria is widely believed to be a hallmark of GS that distinguishes it from Barter’s syndrome, presenting as hypercalciuria. The pathomechanism of hypocalciuria in GS is not fully elucidated. Up to date, a clinical course of GS with normocalciuria has been reported only in men, while women have a milder course of the disease with typical hypocalciuria, which is believed as the result of sex hormone. Additionally, there is a growing evidence that calcium channels of the distal nephron could be regulated by a variety of hormones, including aldosterone (Aldo).

**Case presentation:**

We present the case of a 28-year-old Caucasian woman with asymptomatic, chronic hypokalemia, hypomagnesemia, hypochloremic alkalosis and normal urinary calcium excretion. A high renin levels with normal concentration of Aldo in serum have also been found. The values of blood pressure were low. Based on genetic studies, two heterozygous mutations in the trans position were confirmed: c.2186G>T (p.Gly729Val) and c.1247G>C (p.Cys416Ser) in the *SLC12A3* gene, which ultimately confirmed the diagnosis of GS.

**Conclusions:**

We report here the first case of genetically confirmed GS manifested as normocalciuria in a Caucasian woman. Thus, our result does not confirm a role of sex hormones on the level of calciuria. Based on the results of normal Aldo concentration despite high renin level in our patient, we hypothesized that Aldo may be connecting with the level of urinary calcium excretion in patients with the GS.

## Background

Gitelman Syndrome (GS), (OMIM: 263800), also referred to as familial hypokalemia-hypomagnesemia, is an autosomal recessive congenital tubulopathy, primarily characterized by hypokalemic metabolic alkalosis with significant hypomagnesemia and low urinary calcium excretion described by Hillel Gitelman in 1966 [1]. Most cases of GS are associated with a biallelic inactivating mutations of the *SLC12A3* gene, located on chromosome 16, which encodes thiazide-sensitive sodium chloride cotransporter (NCCT), located in the apical membrane of the distal convoluted tubule (DCT) [2, 3]. To date, about 250 pathogenic variants have been identified in this gene [4]. The disorder occurs in approximately 1–10 per 40,000 Caucasian patients (heterozygotes 1:100) [4]. Thus, GS is one of the most common genetically determined tubulopathies [2, 3].

According to the consensus developed by KDIGO experts [2], clinical criteria indicating GS diagnosis are chronic hypokalemia below 3.5 mmol/L with inadequately elevated a spot urine potassium to creatinine ratio (uK/Cr) above 2.0 mmol/mmol, metabolic alkalosis, hypomagnesemia below 0.7 mmol/L and elevated fractional urinary magnesium excretion > 4%, decreased a spot urine calcium to creatinine ratio (uCa/Cr) below 0.2 mmol/mmol (< 0.07 mg/mg) in adults and fractional chloride excretion above 0.5%. Moreover, the occurrence of elevated serum renin levels and low or normal blood pressure values are typical. As part of the differential diagnosis, it is also recommended performing an ultrasound examination of the kidneys in order to exclude nephrocalcinosis, nephrolithiasis, malformation of the urinary tract and kidney cysts. On the other hand, clinical features against the diagnosis of GS are the use of thiazides or laxatives, evidence of normokalemia, absence of metabolic alkalosis, low renin levels, uK/Cr ratio below 2.0 mmol/mmol (< 18 mmol/g), high ratio of uCa/Cr above 0.57 mmol/mmol, hypertension, edema, abnormalities in kidney ultrasound, maternal polyhydramnios, occurrence of symptoms before the age of 3.

In clinical practice, GS most often requires differentiation with classical Bartter syndrome (BS). The constellations of the metabolic disturbances found in these tubulopathies are similar to the side effects of diuretics. Regarding GS, it corresponds to the disorders found in patients using thiazide diuretics, while in BS, the symptoms are similar to the use of loop diuretics [5]. In both syndromes, patients present similar metabolic disorders such as hypokalemia, hypomagnesemia and metabolic alkalosis. In typical cases, they are distinguished by the presence of hypercalciuria in BS and hypocalciuria in GS [2, 6]. Clinical manifestations of GS are associated with high phenotypic variability. Even concerning an identical NCCT mutations, clinical features of typical GS (with hypocalciuria) were present in women whereas features of typical BS were present in men (without hypocalciuria). Authors have suspected that differences in sex may explain this phenotype variability [7].

We present, a case of Caucasian woman with genetically confirmed GS and atypical clinical manifestation with normocalciuria.

## Case presentation

**Fig. 1:**
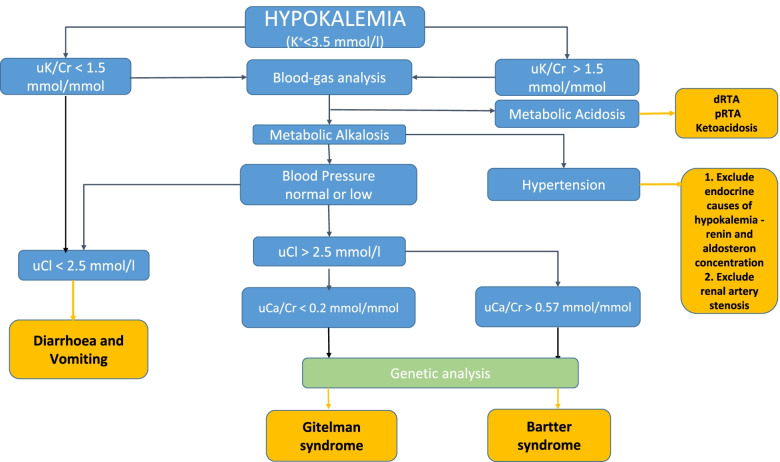
Algorithm for differential diagnosis of hypokalemia

A 28-year-old Caucasian woman was admitted to the Department of Nephrology, Hypertension, and Internal Medicine for the diagnosis of accidentally detected, for the first time in her life, asymptomatic hypokalemia with a potassium concentration of 2.4 mmol/L. In the medical history so far, the patient denied the presence of chronic diseases. Five years prior, she had given birth to a healthy child. The laboratory tests performed at that time had not assessed the concentration of electrolytes in the serum. The patient’s family history was not burdened with kidney and genetic diseases. During hospitalization, the patient did not report any symptoms, and denied using laxatives and diuretics. She had an increased appetite for salted products as well as vegetables and fruits. Physical examination revealed overweight with a BMI of 28 kg/m^2^, without signs of cushingoid body structure and stretch marks. Blood pressure (BP) values were low as 90/60 mmHg. A differential diagnosis of hypokalemia was performed according to the algorithm presented on Fig. 1.

A computed tomography (CT) revealed normal anatomy of adrenal glands. The length of kidneys were 127 mm of right and 103 mm of left. A complete duplication of the renal pelvis was detected. Based on the angio-CT examination, the anatomical variant of the right kidney vascularization by 4 renal arteries was demonstrated, one of which originated from the right common iliac artery, and the others from the aorta. The left kidney had a single renal artery. There was no evidence of hemodynamically significant stenosis or fibromuscular angiodysplasia.

The results of laboratory test regarding urinalysis and blood serum are presented in the following Tables 1, 2 and 3.

**Table 1:** The results of biochemical tests of serum and blood gas analysis at the baseline

Laboratory parameter	Patient results	Normal range
**Biochemical tests of blood serum**		
Sodium [mmol/L]	136.6	136–145
Potassium [mmol/L]	2.4	3.5–5.0
Chloride [mmol/L]	96.6	98–110
Calcium [mmol/L]	2.51	2.1–2.55
Magnesium [mmol/L]	0.55	0.85–1.15
Phosphate [mmol/L]	1.21	0.74–1.52
Serum osmolality [mOsmol/kg H_2_O]	294	285–295
Creatinine concentration [mg/dL]	0.76	0.60–1.10
Glomerular filtration (GFR) according to MDRD [mL/min/1.73m^2^]	96.5	≥90
Renin in supine position [μU/mL]	80.7	4.2–59.7
Renin in upright position [μU/mL]	308.6	5.3–99.1
Aldosterone in supine position [ng/dL]	13.6	3.7–31.0
Aldosterone in upright position [ng/dL]	35.7	3.7–43.2
PTH [pg/mL]	26.2	18,5-88,0
25-hydroxyvitamin D3 [ng/mL]	51.6	30–100
Alkaline phosphatase [U/L]	85	46–116
Glucose [mg/dL]	86	70–99
HbA1c [%]	5.6	< 5,7%
Albumin [g/dL]	4.3	3.5–5.0
Uric acid [mg/dL]	4.8	2.6–6.0
AST [U/l]	21	< 34
ALT [U/l]	34	< 36
**Venous blood gas analysis**		
pH	7.374	7.35—7.45
HCO_3_ concentration [mmol/L]	33	21—27
BE [mmol/L]	5.9	−2.3 — + 2.3
pCO2 [mmHg]	57.8	32—45

**Table 2:** The results of 24-h urine collection at the baseline

Laboratory parameter	Patient result	Normal range
Creatinine excretion [mmol/day]	10.79	6.54–13.88
Sodium excretion [mmol/day]	262.7	40–220
Potassium excretion [mmol/day]	506.2	25–125
Magnesium excretion [mmol/day]	11.7	3.0–5.0
Calcium excretion [mmol/day]	4.32	2.50–8.00
Chloride excretion [mmol/day]	0.45	0.11–0.25
Potassium fractional excretion [%]	79.6	< 6.5% (if hypokalemia)
Sodium fractional excretion [%]	1.2	0–1
Magnesium fractional excretion [%]	12.1	2–4
Calcium fractional excretion [%]	0.12	0.01–0.25
Chloride fractional excretion [%]	3.4	< 0.5

Additionally, an increased concentration of renin has been found with the simultaneous normal Aldo concentration and normal ratio of Aldo to direct renin concentration (ADRR). On this basis, primary hyperaldosteronism was excluded. A normal circadian rhythm of ACTH and cortisol secretion was also demonstrated, as well as the correct concentration of TSH and free thyroid hormones. Immunological studies did not reveal the presence of antinuclear antibodies (ANA) or anti-neutrophil cytoplasm antibodies (ANCA).

**Fig. 2:**
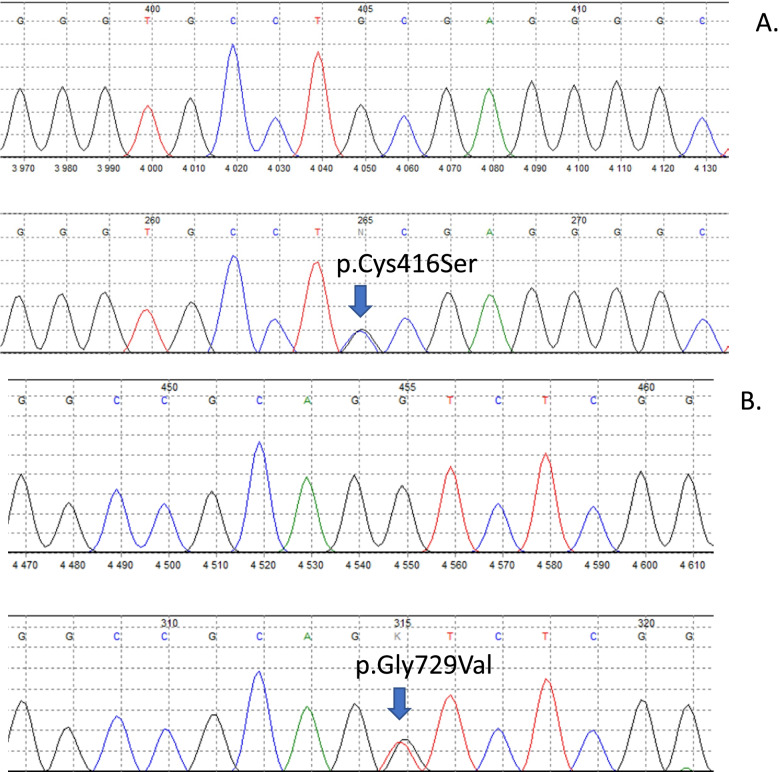
Identification of the SLC12A3 mutations using Sanger sequencing. *Missense mutation c.1247G > C leads to p.Cys416Ser (**A**), mutation c.2186G > T leads to p.Gly729Val (**B**). The reference sequences were: NM_000339.2 and NP_000330.2

A suspicion of tubulopathy was done based on the results of laboratory and imaging tests as well as a clinical manifestation. On the basis of gene sequencing using the Sanger method, the mutation c.2186G > T(p.Gly729Val) in one allele of the *SLC12A3* gene and the coexisting mutation c.1247G > C (p.Cys416Ser) (reference sequences: NM_000339.2 and NP_000330.2) were confirmed (Fig. 2). Both mutations are registered in the ClinVar database as pathogenic variants for Gitelman syndrome (ID: 992415 and 994,770 respectively). Unfortunately, due to the lack of consent of the patient’s parents to conduct genetic tests, it was not possible to determine the exact mechanism of inheriting the mutation.

Analysis was performed with Mutation Surveyor software. The mutants are marked with arrows.

The patient was recommended an oral supplementation of potassium chloride (6 tablets of 600 mg every 4 h) and magnesium chloride (2 tablets of 100 mg every 8 h) as well as a potassium-rich diet. Treatment with mineralocorticoid receptor antagonists (MCRA) or thiazide diuretics were not introduced into therapy due to the planned pregnancy.

## Discussion and conclusions

The differential diagnosis of hypokalemia with hyperkaliuria should include the blood gas analysis and blood pressure measurement, according to the algorithm presented in Fig. 1. The late age of symptoms onset and low blood pressure values, despite the elevated concentration of renin in the serum, as well as characteristic deviations in electrolyte and acid-base in our patient suggested the diagnosis of GS. Although, doubts were raised by the level of urinary calcium excretion, which was in the normal reference range (from 2.52 to 4.32 mmol/day). According to KDIGO, the assessment of electrolyte excretion in spot urine as a ratio to creatinine is recommended. The threshold for diagnosis of hypocalciuria in adults according to this criterion was defined as the uCa/Cr ratio < 0.2 mmol/mmol (< 0.07 mg/mg) [2]. In the analyzed case, the uCa/Cr ratio was 0.39 mmol/mmol, and it was either in the normal laboratory range. What’s more, the authors of the KDIGO consensus has concluded that the levels of calciuria in GS may be variable, and hypomagnesemia does not always occur, therefore only a confirmation of hypercalciuria can exclude the GS [2]. Since, urinary calcium excretion may not always be a reliable test in the differential diagnosis to distinguish GS patients from BS patients, the genetic testing remains the decisive tool in the diagnosis [3].

The medical literature describing the GS cases without hypocalciuria are sparse. The retrospective study conducted on the group of 117 GS patients with confirmed *SLC12A3* mutations has not shown a hypocalciuria (uCa/Cr < 0.04 mg/mg) and hypomagnesemia (< 1.7 mg/dL) in as much as 6 and 8% of patients, respectively [8]. There are also a two case reports available on this topic. The first one describes a female with genetically confirmed GS presenting with pseudo-normal uCa excretion due to coexisting primary hyperparathyroidism (PHP). Surgical removal of the parathyroid gland adenoma has resulted in developing of typical hypocalciuria [9]. We excluded PHP in our patient, on the base of normal levels of serum PTH, calcium and phosphate. Furthermore, the results of serum levels of vitamin D and alkaline phosphatase were normal (Table 1). The second report of GS with normocalciuria concerns two unrelated Chinese families with molecularly proven GS, in which male patients had severe hypokalemia with episodes of paralysis from childhood, impaired maximal urine concentrating ability, normal serum Mg and normal uCa excretion (laboratory findings typical of BS) [7]. In opposite, female patients were asymptomatic. They had milder hypokalemia, the intact urine concentration ability, hypomagnesemia and hypocalciuria (laboratory findings typical of GS). Nevertheless, all patients had the same novel pair of *SLC12A3* mutations – an adenosine to cytosine single base substitution at nucleotide 2135 (C2135A, TCG to TAG) on 1 allele and 2 base pair deletions at nucleotide 2881–2 (del AG) on the second allele were found in exon 17 and exon 24, respectively [7]. In our report, the course of GS was mild and oligosymptomatic, and there was no hypocalciuria. A genetic study confirmed the presence of two missense mutations in the *SLC12A3* gene: c.2186G > T (p.Gly729Val) and c.1247G > C (p.Cys416Ser). Interestingly, in a different report presenting a 6-year-old boy of Polish origin with GS secondary to an identical SLC12A3 mutations c.2186G > T (p.Gly729Val) and c.1247G > C (p.Cys416Ser) the presence of hypocalciuria has been indicated [10]. Thus, these results do not prove the role of specific mutations nor sex hormones as a contributory factors for hypocalciuria in GS. Although, the sex hormone may have an impact on the severity of disease and the age of symptom onset.

The renal excretion of Ca is precisely regulated in the DCT and CNT, which actively reabsorb about 10% of the filtered Ca load under normal conditions. In these segments, Ca reabsorption is mediated by transient receptor potential vanilloid 5 (TRPV5) and 6 (TRPV6) channels localized on luminal membrane [11]. The pathomechanism of hypocalciuria in GS is not fully elucidated yet, but it can be connected with the primary defect of NCCT as this disorder is observed after administration of thiazide diuretics which inhibit the NCCT and in the model of NCCT deficient mice [12]. The NCCT defect is believed to be responsible for the decreased reabsorption of NaCl. The increased NaCl load that reaches further part of the DCT and the collecting duct (CD) is responsible for the excessive loss of water. Secondary hypovolemia stimulates increased renin secretion from the juxtaglomerular apparatus and leads to the activation of the renin-angiotensin-aldosterone system (RAA). Aldo by stimulating epithelial Na channels (ENaC) in the apical plasma membrane of the DCT and the CD leads to increased reabsorption of Na ions, which is counterbalanced by the simultaneous excretion of K ions into the urine through ROMK (renal outer-medullary potassium) channels. Na reabsorption also produces an electrochemical gradient that facilitates the excretion of hydrogen (H) ions by the alpha intercalated cells in the DCT and the CT, leading to the development of metabolic alkalosis [13]. Aldo also increases the magnitude of the lumen negative transepithelial voltage and decline the intracellular Cl concentrations, which hyperpolarizes the plasma membrane of DCT cells [14].

Hyperpolarization of the luminal membrane of the DCT cells stimulate the entry of Ca into the cell through TRPV5 and TRPV6 channels [15]. In turn, increased activity of the type 1 Na/Ca exchangers (NCX1) and an ATP-dependent Ca pumps (PMCA1b) in the basolateral membrane are responsible for the transport into the blood compartment of 70 and 30% of Ca, respectively [16]. Additionally, a lumen-negative transepithelial voltage favors Mg secretion [14].

Aldo physiologically increases the expression of the ENaC channels in the apical plasma membrane and the activity of NCCT in the DCT [17]. The elevated Aldo levels also play a role in the adaptive changes seen in the distal nephron of NCCT – deficient (NCC −/−) animals. The ultrastructural analysis performed in NCC −/− mice (an animal model of GS) shows that the early segment of DCT (DCT1), which physiologically lacks epithelial ENaC and TRPV5 channels, is almost absent. While the late segment of DCT (DCT2) seems intact and shows a weak expression of ENaC and a high expression of TRPV5. In contrast, the connecting tubule (CNT) has exhibited a marked epithelial hypertrophy accompanied by an increased apical abundance of ENaC. Thus, increased Na reabsorption through ENaC in the CNT of NCC −/− mice may be an adaptation mechanism stimulated by the elevated plasma Aldo levels [18]. There is a growing evidence that Aldo despite Na also enhances Ca reabsorption by the distal nephron [19]. An experimental study shows that the incubation of rabbit distal tubules with Aldo enhances transport of both Na and Ca by the luminal membranes. Aldo increases the number of Ca channels in the plasma membranes without influencing their affinity for Ca. What’s more, the effect of Aldo on Ca transport has been inhibited by addition of NaCl in concentration of 100 mmol/L into the kidney tubules [20]. Similarly to Aldo, the most of the other Ca regulating hormones, such as PTH, calcitonin or Ang II act both on the Na and Ca cation transports in the distal nephron but in opposite directions [20].

Additionally, it has been shown in GS patient that uCa excretion increased from 0.2 to 3.4 mmol/day during a high-sodium diet (250 mmol/day) period, while at the same time uNa excretion increased from 12 to 239 mmol/day, respectively [21].

In the presented case, the highest values of the uCa/Cr ratio corresponded to the highest concentration of Na in a spot urine. Moreover, there was an inverse relationship between the level of Na and Ca in the urine and the concentration of Aldo in the serum (Table 3).

**Table 3:** The results of biochemical tests of spot urine and serum at the baseline and the control visit

Laboratory parameter	Patient result at the baseline	Patient result at the control	Normal range
**Biochemical tests of spot urine**			
pH	8	7.5	5.0–6.9
Urine-specific gravity	1.008	1.016	> 1.023
Protein	negative	negative	negative
Albumin to creatinine ratio (ACR) [mg/mmol]	0.752	Not done	< 3
Protein to creatinine ratio (PCR) [mg/mmol]	14.2	21.3	< 15
Glucose [mg/dL]	absent	absent	(−)
Urine osmolality [mOsmol/kg H_2_O]	454	Not done	50–1400
Sodium concentration [mmol/L]	65	41.5	25–150
Potassium concentration [mmol/L]	121.8	Not done	25–100
Chloride concentration [mmol/L]	165	306	20–40
Magnesium concentration [mmol/L]	3.29	6.30	0.4–6.67
Calcium to creatinine ratio (Ca/Cr) [mmol/mmol]	0.39	0.24	0.20–0.57
**Biochemical tests of blood serum**			
Aldosterone in supine position	35.7	52.0	3.7–43.2
Creatinine concentration [mg/dL]	0.67	0.57	0.60–1.10
Glomerular filtration rate (GFR) according to CKD-EPI [mL/min/1.73m^2^]	120	124	≥90
Sodium [mmol/L]	139.2	140.9	136–145
Potassium [mmol/L]	3.9	3.9	3.5–5.0
Chloride [mmol/L]	98.8	104.9	98–110
Calcium [mmol/L]	2.42	2.42	2.1–2.55
Magnesium [mmol/L]	0.53	0.62	0.85–1.15

According to the other hypothesis, hypocalciuria in GS ca be the result of mild contraction of the extracellular fluid volume and subsequent increased Ca reabsorption in the proximal tubule [22]. Dietary Na restriction has been reported to aggravate the volume depletion and to augment the hypocalciuria in NCC-deficient mice [12]. Although, a study conducted in patients with GS, who received an infusion of isotonic saline, has not confirmed that hypovolemia is the main factor responsible for hypocalciuria [23]. On the other hand, hypovolemia and salt wasting in GS are responsible for stimulation of increased levels of serum renin and Aldo.

Surprisingly, the results of our patient’s confirmed a normal Aldo concentration despite significantly increased level of renin. At the same time, the correct responses of renin and Aldo were demonstrated in functional tests (Table 1). It has been proven that serum Aldo may not be as high as expected for the degree of hyperreninemia in BS and GS due to a low total body potassium content [21]. Additionally, an impairment of chymase-dependent conversion of angiotensin (Ang) I to Ang II has been responsible for normal levels of Ang II and Aldo despite a few times greater than normal concentrations of renin and Ang I in GS patient [19]. Moreover, the study which analyzed the effect of diet on RAA axis in GS patient has shown that concentration of Aldo and plasma renin activity (PRA) increases during the low-sodium and high-potassium diets. While a high-sodium diet does not inhibit PRA, it decreases Aldo level. There have also been normal responses to posture with elevation in PRA and Aldo on ambulation compared to recumbent values [21].

In conclusion, we believe that in the presented case of a patient with Gitelman’s syndrome, normocalciuria could depend on the hydration status, dietary sodium content, as well as a low aldosterone levels (Table 3). Finally, we cannot exclude that structural damage to DCT secondary to prolonged hypokalemia in GS also contributed to calcium leakage. Further, studies in larger groups of patients with GS are necessary to confirm this observation.

## Data Availability

The authors declare that [the/all other] data supporting the findings of this study are available within the article [and its supplementary information files].

## Abbreviations

ADRR: A ratio of aldosterone to direct renin concentration
Aldo: Aldosterone
ANA: Antinuclear antibodies
ANCA: Anti-neutrophil cytoplasm antibodies
BS: Barter syndrome
CLC-KB: Kidney-specific basolateral chloride channel
CT: A computed tomography
dRTA: Distal tubular renal acidosis.
ENaC: Epithelial sodium channel
GS: Gitelman syndrome
NaCl: Sodium chloride
NCCT: Thiazide-sensitive sodium chloride cotransporter
NCX1: Type 1 of Na/Ca exchanger
MCRA: Mineralocorticoid receptor antagonists
PHP: Primary hyperparathyroidism
PMCA: Plasma membrane calcium ATPase
pRTA: Proximal renal tubular acidosis
ROMK: Renal outer-medullary potassium channels
TRPM6: The transient receptor potential cation channel subfamily M member 6
TRPV5: The apical transient receptor potential vanilloid 5 calcium channel
TRPV6: Transient receptor potential vanilloid subfamily member 6
uCa: Urinary calcium excretion
uCa/Cr: A spot urine calcium to creatinine ratio
uCl: Urinary chloride excretion
uK/Cr: A spot urine potassium to creatinine ratio
uNa: Urinary sodium excretion
